# A National Public Health Establishment

**Published:** 1895-01

**Authors:** 


BUFFALO MEDICAL AND SURGICAL JOURNAL

A MONTHLY REVIEW OF MEDICINE AND SURGERY.

*EDITORS:*

THOMAS LOTHROP, M. D. - - WM. WARREN POTTER, M. D.

All communications, whether of a literary or business nature, should be addressed to the managing editor: 284 FRANKLIN STREET, BUFFALO, N. Y.

VOL. XXXIV. JANUARY, 1895. No. 6.

### A NATIONAL PUBLIC HEALTH ESTABLISHMENT.

THE propriety of placing the supervision of the public health in the hands of the general government has been discussed with much freedom of late, both in the columns of the professional and lay press. Though the JOURNAL has not taken an active part in the discussion, it has not been, nor is it, indifferent to the subject. We recognize the importance of placing the control of public health problems in the hands of national authority. Moreover, we are of the opinion that such authority should be vested in a cabinet officer appointed to preside over a department of health. There is every argument in favor of such a plan. Foreign governments have adopted it. Powers of much less importance than the United States create ministers of health who preside over a department.

Here in the United States we do not hesitate to create a department of justice with an attorney-general at its head and subordinate attorneys scattered throughout the several districts and who are responsible to the head of the department at Washington. Then, too, there is a supreme court with all its necessary judges, clerks, attorneys, solicitors and other officers. All this machinery is necessary to properly administer the law department of the government.

Why should not the paramount questions pertaining to public health be committed to a similar department presided over by a minister who is an educated physician and an expert in public sanitation?

Is there not a greater reason why the general government should protect health and life than for it to guard its legal interests with such scrupulous care? The economics of the one are quite as essential to progression and improvement as the other, and in our opinion even more so.

In this connection we desire to support the recommendation of the President in his recent message to Congress, wherein he suggests the establishment of a national board of health, or of a national health officer, to control matters pertaining to the protection of the country against epidemic, infectious and pestilential disease.

If such an office or board should be created, it would be a long stride in the direction which we advocate. It is not, to be sure, as completely planned as we could wish, but we shall give such a proposition, if it takes the form of a bill before Congress, our cordial commendation.

